# Melatonin Suppresses Autophagy Induced by Clinostat in Preosteoblast MC3T3-E1 Cells

**DOI:** 10.3390/ijms17040526

**Published:** 2016-04-08

**Authors:** Yeong-Min Yoo, Tae-Young Han, Han Sung Kim

**Affiliations:** 1 Yonsei-Fraunhofer Medical Device Laboratory, Department of Biomedical Engineering, Yonsei University, Wonju, 26493 Gangwon-do, Korea; yyeongm@hanmail.net; 2 Fraunhofer Institute IKTS-MD, Maria-Reiche-Str.2, 01109 Dresden, Germany; tae-young.han@ikts-md.fraunhofer.de

**Keywords:** melatonin, autophagy, ERK, Akt, mTOR, clinostat, MC3T3-E1 cells

## Abstract

Microgravity exposure can cause cardiovascular and immune disorders, muscle atrophy, osteoporosis, and loss of blood and plasma volume. A clinostat device is an effective ground-based tool for simulating microgravity. This study investigated how melatonin suppresses autophagy caused by simulated microgravity in preosteoblast MC3T3-E1 cells. In preosteoblast MC3T3-E1 cells, clinostat rotation induced a significant time-dependent increase in the levels of the autophagosomal marker microtubule-associated protein light chain (LC3), suggesting that autophagy is induced by clinostat rotation in these cells. Melatonin treatment (100, 200 nM) significantly attenuated the clinostat-induced increases in LC3 II protein, and immunofluorescence staining revealed decreased levels of both LC3 and lysosomal-associated membrane protein 2 (Lamp2), indicating a decrease in autophagosomes. The levels of phosphorylation of mammalian target of rapamycin (p-mTOR) (Ser2448), phosphorylation of extracellular signal-regulated kinase (p-ERK), and phosphorylation of serine-threonine protein kinase (p-Akt) (Ser473) were significantly reduced by clinostat rotation. However, their expression levels were significantly recovered by melatonin treatment. Also, expression of the Bcl-2, truncated Bid, Cu/Zn- superoxide dismutase (SOD), and Mn-SOD proteins were significantly increased by melatonin treatment, whereas levels of Bax and catalase were decreased. The endoplasmic reticulum (ER) stress marker GRP78/BiP, IRE1α, and p-PERK proteins were significantly reduced by melatonin treatment. Treatment with the competitive melatonin receptor antagonist luzindole blocked melatonin-induced decreases in LC3 II levels. These results demonstrate that melatonin suppresses clinostat-induced autophagy through increasing the phosphorylation of the ERK/Akt/mTOR proteins. Consequently, melatonin appears to be a potential therapeutic agent for regulating microgravity-related bone loss or osteoporosis.

## 1. Introduction

Microgravity induces physiological and environmental stresses on animals, including humans. Microgravity exposure can cause cardiovascular and immune disorders, muscle atrophy, osteoporosis, and loss of blood and plasma volume [1,2,3,4]. Techniques have been developed to research the effects of microgravity by simulating microgravity on Earth [5]. A clinostat is an effective ground-based tool for simulating microgravity [6,7] and has been used to study the impact of microgravity on cells and tissues [8,9,10,11] in studies aimed at evaluating microgravity in cell biology, tissue engineering, and biomedical engineering [5,12]. In particular, tissue engineering studies have evaluated the formation of three-dimensional (3D) multicellular structures for use in cartilage regeneration and for the construction of artificial vessel and cancer spheroids. Other studies have evaluated the molecular and cellular mechanisms of angiogenesis, cancer development, and stem cell biology. Hence, these studies can help in the development of new strategies for medical transplantation and regenerative medicine [5,12].

Microgravity during space flight leads to decreases in the mineral content of bone, bone matrix protein production and bone formation, resulting in bone loss and osteoporosis [13,14]. A clinostat can be used to provide valuable information about mechanotransduction as it relates to bone homeostasis in bone cells, as well as for *in vitro* mechanobiology studies in the bone microenvironment [14,15]. Maintenance of bone homeostasis is based on the regulation of biochemical responses through balancing the activities of osteoblasts, osteoclasts, and osteocytes based on mechanosensitive signal transduction from microenvironmental forces, including mechanostimulation and mechanical stress. Despite the significant progress made in studies under microgravity and simulated conditions, the signal transduction mechanism of mechanotransduction in bone cells is still not well defined.

Several studies have proposed that bone loss may be a consequence of decreased osteoblast viability caused by the induction of apoptosis in microgravity [16,17,18,19,20,21]. Conversely, an additional study showed that osteoblast apoptosis was not induced by simulated microgravity, suggesting that microgravity does not directly induce osteoblast death [22]. Microgravity may result in increased osteoclast activity, thus potentially contributing to bone loss [23,24,25,26]. A recent study reported that microgravity-induced autophagy plays an important role in enhanced osteoclast differentiation and may be a potential therapeutic target to prevent bone loss [27].

Melatonin, a hormone secreted from the pineal gland of the brain, has an anti-apoptotic effect as an antioxidant molecule and a suppressive function on autophagy [28,29,30,31,32,33,34]. Qu *et al.* [35] reported that melatonin protects PC12 cells from oxidative damage during simulated weightlessness. Evidence demonstrating a direct interaction between melatonin and microgravity-induced autophagy has not been reported. In this study, we provide the first demonstration that melatonin reduces autophagy induced by clinostat rotation in preosteoblast MC3T3-E1 cells.

## 2. Results

### 2.1. Autophagy Was Induced by Clinostat Rotation

**Figure 1:**
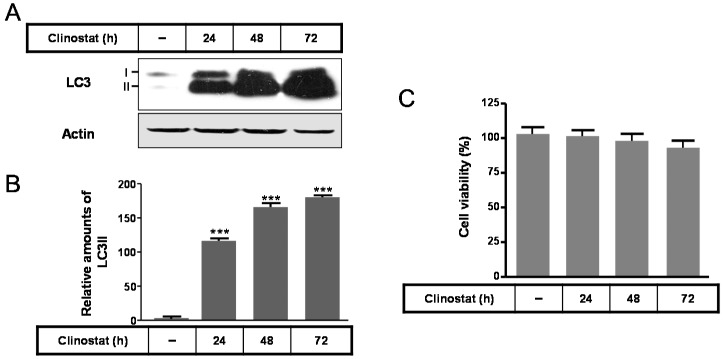
Expression of the microtubule-associated protein light chain (LC3) protein with clinostat rotation in MC3T3-E1 cells. MC3T3-E1 cells were incubated in α-minimum essential medium (α-MEM) added with 10% fetal bovine serum (FBS) and 1% Penicillin-Streptomycin (PS) at 37 °C with 5% CO_2_. To experimentally simulate microgravity with the 3D clinostat, cells were cultured in T-25 cell culture flasks completely filled with a 1:1 mixture of α-MEM and Leibovitz’s L-15 supplemented with 10% FBS and 1% PS. LC3 expression was then performed by Western blot analysis (**A**); The relative expression of LC3 (**B**) was quantified and cell survival (**C**) was carried out as described in the Materials and Methods section. Values in representative results are shown as means ± SD of at least three independent experiments. *** *p* < 0.001 *vs.* no clinostat.

In preosteoblast MC3T3-E1 cells, the expressions of the autophagosomal or autophagy marker protein microtubule-associated protein light chain (LC3) II significantly increased in a time-dependent manner by clinostat rotation (Figure 1A,B) and cell survival did not appeared significant (Figure 1C). This suggests that the autophagy in preosteoblast MC3T3-E1 cells was induced by clinostat rotation without any decrease in cell viability.

### 2.2. Melatonin Attenuates Autophagy by Clinostat Rotation

**Figure 2:**
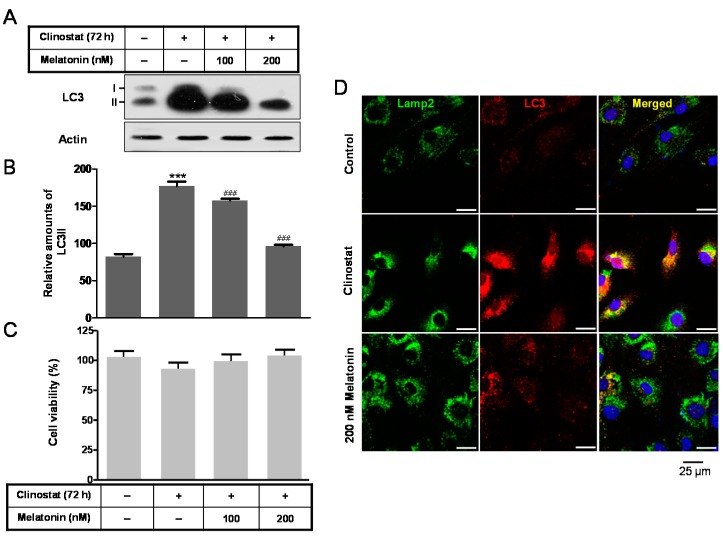
LC3 expression with clinostat rotation and/or melatonin treatment in MC3T3-E1 cells. MC3T3-E1 cells were cultured in α-MEM added with 10% FBS at 37 °C with 5% CO_2_. To experimentally simulate microgravity with the 3D clinostat, cells were cultured in T-25 cell culture flasks completely filled with a 1:1 mixture of α-MEM and Leibovitz’s L-15 supplemented with 10% FBS and 1% PS, with or without melatonin (100, 200 nM). LC3 expression was then performed by Western blot analysis (**A**); the relative expression of LC3 (**B**) was quantified as described in the Materials and Methods section. Immunofluorescence levels of both LC3 and Lamp2 indicate the degree of autophagosomes or autophagy; Cell survival (**C**) was measured and immunofluorescence analysis of LC3, Lamp2; and DAPI staining (**D**) was carried out as described in Materials and Methods. Values in representative results are shown as means ± SD of at least three independent experiments. (**D**) Scale bars, 25 µm. *** *p* < 0.001 *vs.* No clinostat. ### *p* < 0.001 *vs.* clinostat.

To identify the effect of melatonin, the addition of 100 and 200 nM of melatonin to clinostat-treated cells significantly attenuated the clinostat-induced increase in LC3 II protein (Figure 2A,B). Quantitative immunofluorescences of both LC3 and lysosomal-associated membrane protein 2 (Lamp2) mean the degree of autophagosomes or autophagy. In this condition, cell survival did not appear significant (Figure 2C). Immunofluorescence staining of cells treated with 200 nM melatonin was carried out to detect the colocalization of LC3 and Lamp2. The clinostat-increased LC3-positive granules or puncta were colocalized with the increased immunofluorescences of Lamp2. Melatonin treatment reduced this colocalization, indicating that autophagosomes or autophagy decrease under conditions of both melatonin treatment and clinostat rotation (Figure 2D).

### 2.3. Melatonin Increases Levels of p-mTOR, p-ERK and p-Akt Proteins

**Figure 3:**
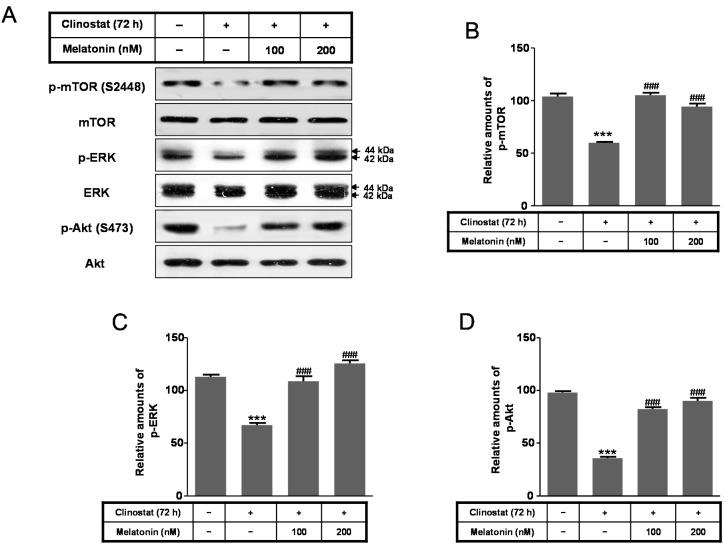
Phosphorylation of mTOR, ERK, and Akt with clinostat rotation and/or melatonin treatment in MC3T3-E1 cells. MC3T3-E1 cells were cultured in α-MEM added with 10% FBS at 37 °C with 5% CO_2_. To experimentally simulate microgravity with the 3D clinostat, cells were cultured in T-25 cell culture flasks completely filled with a 1:1 mixture of α-MEM and Leibovitz’s L-15 supplemented with 10% FBS and 1% PS, with or without melatonin (100, 200 nM). Phosphorylation of mTOR, ERK, and Akt was then performed by Western blot analysis (**A**); the relative expressions of p-mTOR (**B**); p-ERK (**C**); and p-Akt (**D**) were carried out as described in the Materials and Methods section. Values in representative results are shown as means ± SD of at least three independent experiments. *** *p* < 0.001 *vs.* No clinostat; ### *p* < 0.001 *vs.* clinostat.

Using the same conditions, markers of cell survival/proliferation signaling pathways, including phosphorylation of extracellular signal-regulated kinase (p-ERK), phosphorylation of serine-threonine protein kinase (p-Akt), and phosphorylation of mammalian target of rapamycin (p-mTOR), were evaluated. Levels of p-mTOR (Ser 2448), p-ERK, and p-Akt (Ser473) were significantly reduced by clinostat rotation; however, their expression levels were significantly recovered by melatonin treatment in conjunction with clinostat rotation (Figure 3). These results indicated that the microgravity condition in melatonin and clinostat-treated cells positively regulates the phosphorylation of ERK/Akt/mTOR.

### 2.4. Melatonin Increases Levels of Bcl-2, Truncated Bid, Cu/Zn-SOD, Mn-SOD GRP78/BiP, IRE1α and p-PERK Proteins

**Figure 4:**
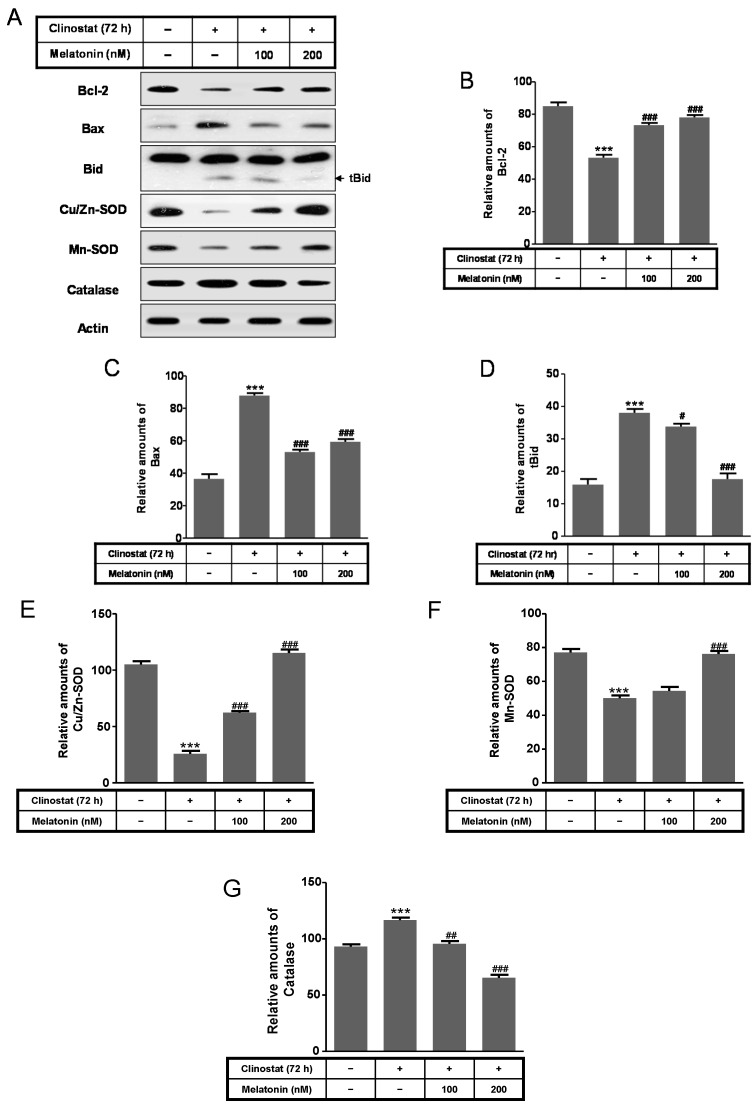
Expression of Bcl-2, Bax, truncated Bid (tBid), Cu/Zn-SOD, Mn-SOD, and catalase with clinostat rotation and/or melatonin treatment in MC3T3-E1 cells. MC3T3-E1 cells were cultured in α-MEM added with 10% FBS at 37 °C with 5% CO_2_. To experimentally simulate microgravity with the 3D clinostat, cells were cultured in T-25 cell culture flasks completely filled with a 1:1 mixture of α-MEM and Leibovitz’s L-15 supplemented with 10% FBS and 1% PS, with or without melatonin (100, 200 nM). Phosphorylation of mTOR, ERK, and Akt was then performed by Western blot analysis (**A**); The relative expressions of Bcl-2 (**B**); Bax (**C**); truncated Bid (tBid) (**D**); Cu/Zn-SOD (**E**); Mn-SOD (**F**); and catalase (**G**) were carried out as described in the Materials and Methods section. Values in representative results are shown as means ± SD of at least three independent experiments. *** *p* < 0.001 *vs.* No clinostat; # *p* < 0.05, ## *p* < 0.01, ### *p* < 0.001 *vs.* clinostat.

**Figure 5:**
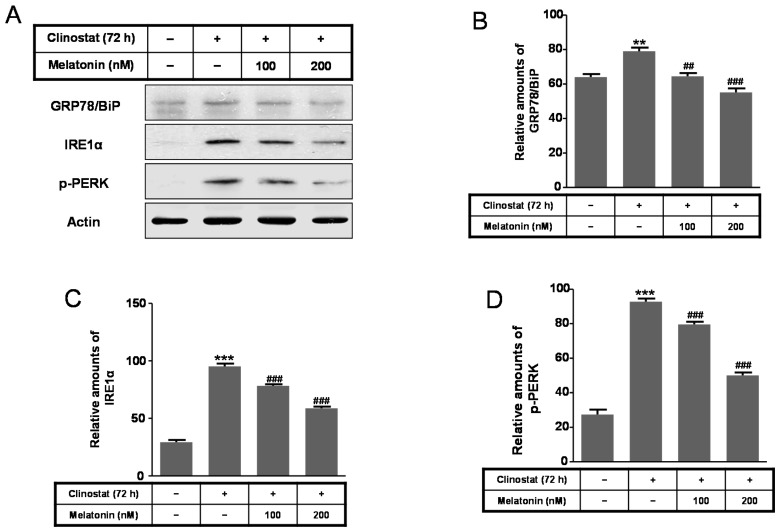
Expression of GRP78/BiP, IRE1α, and p-PERK with clinostat rotation and/or melatonin treatment in MC3T3-E1 cells. MC3T3-E1 cells were cultured in α-MEM added with 10% FBS at 37 °C with 5% CO_2_. To experimentally simulate microgravity with the 3D clinostat, cells were cultured in T-25 cell culture flasks completely filled with a 1:1 mixture of α-MEM and Leibovitz’s L-15 supplemented with 10% FBS and 1% PS, with or without melatonin (100, 200 nM). Phosphorylation of GRP78/BiP, IRE1α, and p-PERK was then performed by Western blot analysis (**A**); The relative expressions of GRP78/BiP (**B**); IRE1α (**C**); and p-PERK (**D**) were carried out as described in the Materials and Methods section. Values in representative results are shown as means ± SD of at least three independent experiments. ** *p* < 0.01, *** *p* < 0.001 *vs.* No clinostat; ## *p* < 0.01, ### *p* < 0.001 *vs.* clinostat.

Next, to characterize intracellular and mitochondrial conditions based on antioxidant and pro-oxidant proteins, we examined how melatonin affected the superoxide dismutase (SOD), catalase, Bcl-2, Bid, and Bax proteins in preosteoblast MC3T3-E1 cells in the clinostat. Expression of Bcl-2, truncated Bid (tBid), Cu/Zn-SOD, and Mn-SOD increased significantly in melatonin-treated clinostat conditions, whereas the levels of Bax and catalase decreased (Figure 4). Levels of the endoplasmic reticulum (ER) stress marker GRP78/BiP, IRE1α, and p-PERK were significantly reduced in melatonin-treated clinostat samples (Figure 5).

### 2.5. Inactivation of Autophagy by Melatonin Is Directly Linked to the Receptor-Mediated Actions

**Figure 6:**
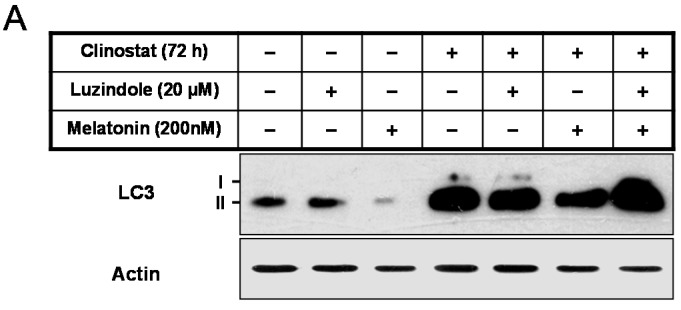

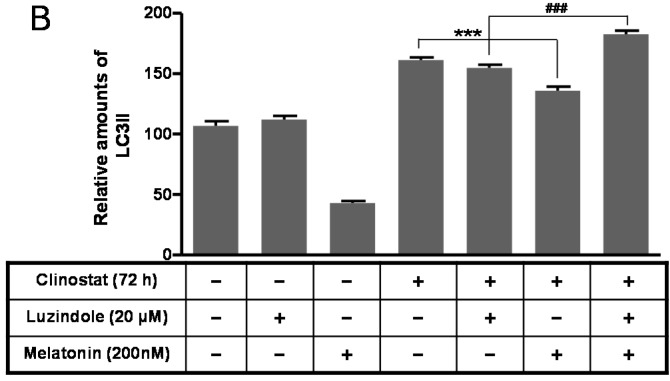
The expression of the LC3 protein with clinostat rotation and/or melatonin treatment and luzindole, melatonin receptor antagonist, in MC3T3-E1 cells. MC3T3-E1 cells were cultured in α-MEM added with 10% FBS at 37 °C with 5% CO_2_. To experimentally simulate microgravity with the 3D clinostat, cells were cultured in T-25 cell culture flasks completely filled with a 1:1 mixture of α-MEM and Leibovitz’s L-15 supplemented with 10% FBS and 1% PS, with or without melatonin (200 nM) and/or luzindole (20 µM). LC3 expression was then performed by Western blot analysis (**A**); the relative expression of LC3 (**B**) was carried out as described in the Materials and Methods section. Values in representative results are shown as means ± SD of at least three independent experiments. *** *p* < 0.001, clinostat *vs.* clinostat and melatonin; ### *p* < 0.001, clinostat and luzindole *vs.* clinostat, melatonin, and luzindole.

The physiological role of melatonin could be either dependent or independent of its receptor-mediated actions. To examine the involvement of melatonin receptors in clinostat-induced autophagy, melatonin receptor antagonist luzindole was used in combination with melatonin. Luzindole abolished the melatonin-induced decrease in LC3 II levels (Figure 6). Thus, inactivation of autophagy with melatonin is directly linked to the receptor-mediated actions of melatonin in preosteoblast MC3T3-E1 cells.

## 3. Discussion

**Figure 7:**
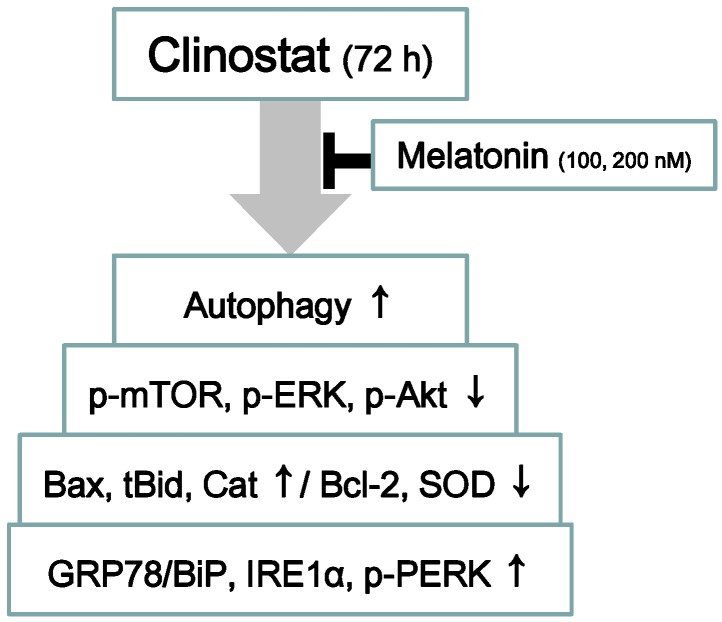
The summarized results above indicate that melatonin suppresses clinostat-induced autophagy through increasing the phosphorylation of the ERK/Akt/mTOR proteins. ↑, Activation or increase of protein level; ↓, Decrease of protein level; ├, Inhibition or suppression.

The present study reported that the preosteoblast MC3T3-E1 cells treated with melatonin suppresses clinostat-induced autophagy with increased phosphorylation of the ERK/Akt/mTOR proteins and that inactivation of autophagy with melatonin treatment directly involves the receptor-mediated actions of melatonin (Figure 7).

Use of the clinostat resulted in a continuous activation of autophagy in preosteoblast MC3T3-E1 cells which were sustained up to 5 days with increased levels of LC3 II protein (Figure 1, data not shown). Under these conditions of simulated microgravity, cell proliferation increased for 3 days and resulted in cell confluence in T-25 cell culture flasks for 5 days and subsequent cell death (not shown data). Strategies to regulate autophagy in tumor cells are promising avenues for novel cancer therapies [36,37]. Downregulated expression of maternally expressed gene 3 (MEG3), encoding a long non-coding RNA associated with tumorigenesis, activates autophagy and increases cell proliferation in bladder cancer tissues [38], suggesting that maintenance of cell survival by autophagy can promote the growth of established tumors. This report and our study indicate that activation of autophagy through tumor-regulatory genes or a simulated microgravity condition is predominantly cytoprotective in tumor cells, and that the suppression of autophagy can enhance tumor cell death in a manner similar to anticancer therapies. Other strategies for activating autophagy in tumor cells are based on more general attempts to suppress cancer cells or stimulate cancer cell death [36,37]. Several studies have shown that cancer cells exposed to autophagy inducers exhibit upregulation of autophagic activity resulting in suppression of tumor progression in human lung cancer, colorectal cancer, and breast cancer cells [39,40,41,42]. With respect to maintenance of bone homeostasis, Sambandam *et al.* [27] demonstrated that autophagy induced by simulated microgravity modulates osteoclastogenesis and may regulate bone loss. Therefore, induction of autophagy as a novel therapeutic strategy presents a new opportunity for cancer suppression as well as for bone regulation [37].

The present study demonstrated that treatment of cells with a physiological nanomolar concentration of melatonin combined with clinostat rotation significantly reduced clinostat-increased autophagy in preosteoblast MC3T3-E1 cells (Figure 2). The antioxidant melatonin is known to regulate autophagy involved with oxidative stress, ER stress, and mitochondria dysfunction [31,32,33,34,35,43,44,45]. Regulation of autophagy by several protein kinases including mTOR, Akt, and ERK, is an important therapeutic target for promoting cell survival and preventing cancers and other diseases [46,47]. In this present study, reductions in p-mTOR, p-ERK, and p-Akt caused by simulated microgravity condition were significantly recovered by applying melatonin treatment in preosteoblast MC3T3-E1 cells (Figure 3).

Of interest, these kinases, including mTOR, Akt, and ERK, have been reported to mediate autophagy in response to ER stress. The amassment of unfolded and misfolded proteins in the ER lumen leads to the unfolded protein response (UPR) and induces autophagy, enabling promotion of the elimination of unfolded or misfolded proteins [47,48]. These ER stress-activated molecules, including GRP78/BiP, IRE1, and PERK, are associated with activation of autophagy. Once activated, these proteins can ultimately result in the upregulation of autophagy signaling. However, reductions in ER stress and protection from autophagy have been observed after melatonin treatment [43,49,50,51]. Autophagy was increased during oxidative stress induced by cyclosporine, whereas it was suppressed by treatment with melatonin [45]. This study showed that the simulated microgravity condition increases the levels of GRP78/BiP, IRE1α, and p-PERK, which are significantly reduced in melatonin-treated preosteoblast MC3T3-E1 cells.

The Bcl-2 family proteins function as dual regulators of apoptosis and autophagy and are consequently involved in both inhibition and induction of autophagy. This ability of the Bcl-2 family proteins to regulate interactions between autophagy and apoptosis plays an important role in development, cellular homeostasis, oncogenesis, and tumor suppression/progression [52,53]. Bcl-xL and Bcl-2 are anti-apoptotic and anti-autophagic proteins, which distributes in the mitochondrial and ER membranes. For example, Bcl-2 inhibits autophagy through Ca^2+^ release from the ER to the cytosol, indicate that Bcl-2 decreases the free Ca^2+^ concentration within the ER [54]. Bcl-xL and Bcl-2 inhibit apoptosis by binding to the pro-apoptotic proteins Bax or Bak. Bcl-2 and Bcl-xL suppress autophagy by binding to Beclin 1, which is required for the initiation of autophagosome formation in autophagy. Thus, Bcl-2 and Bcl-xL help cells escape autophagic cell death [52,53]. Our last study confirmed that melatonin protects against apoptotic cell death by nitric oxide and autophagic cell death by serum starvation in C2C12 murine myoblast cells. We reported that melatonin functions to decrease pro-apoptotic Bax, increase anti-apoptotic Bcl-2, and decrease the Bax/Bcl-2 relative ratio, in C2C12 cells [31]. Clinostat rotation increased the Bax/Bcl-2 ratio in normal human osteoblastic cells [20]; however, the present study demonstrated that melatonin increased Bcl-2 and decreased Bax protein levels in simulated microgravity conditions using preosteoblast MC3T3-E1 cells.

Reactive oxygen species (ROS) are highly reactive molecules and include singlet oxygen, hydroxyl radicals, superoxide, and hydrogen peroxides as byproducts produced from the mitochondrial respiratory chain in cells. ROS are highly reactive against cell constituents, including lipids, proteins, and DNA, thus damaging cell structures and resulting in cell death, apoptosis, necrosis, and autophagy [55,56,57]. Cells can protect against oxidative damage from increases in reactive ROS by alleviating antioxidant enzymes such as the representative redox system, SOD and catalase. This ROS damage has been shown to cause various types of cell death, but whether it plays a role in autophagy has not been evaluated, particularly under conditions of simulated microgravity. The activities of SOD, glutathione peroxidase, and catalase, were all significantly increased 12 h after microgravity onset, yet were decreased at 96 h [58]. The present study showed that simulated microgravity decreased the levels of the Cu/Zn-SOD and Mn-SOD proteins and increased catalase protein levels. However, melatonin treatment acted contrary to the simulated microgravity effect in preosteoblast MC3T3-E1 cells. In addition to the molecular regulatory mechanisms of catalase in autophagic cell death, others may exist in oxidative stress [57].

## 4. Materials and Methods

### 4.1. Cell Culture and Treatment with Melatonin and/or Luzindole

MC3TC-E1 murine preosteoblast cells were cultured in α-Minimum Essential Medium (α-MEM, Gibco BRL, Gaithersburg, MD, USA) added with 10% heat-inactivated fetal bovine serum (FBS, Gibco BRL) and 1% Penicillin-Streptomycin (PS) at 37 °C with 5% CO_2_ in a humidified incubator. To experimentally simulate microgravity with the 3D clinostat, cells were cultured in T-25 cell culture flasks (Falcon, Lincoln Park, NJ, USA) completely filled with a 1:1 mixture of α-MEM and Leibovitz’s L-15 (Invitrogen, Auckland, NZ, USA) supplemented with 10% FBS and 1% PS, with or without melatonin (Sigma-Aldrich, St. Louis, MO, USA) and/or luzindole, melatonin receptor antagonist, (20 µM) (Sigma-Aldrich) and sealed. The culture dishes were completely filled in order to eliminate any possible effects of air bubbles and shear forces.

### 4.2. Three-Dimensional Clinostat (3D Clinostat)

The 3D clinostat (Yonsei University, Wonju, Korea) was developed to simulate microgravity in cells and was designed to fit in a standard cell culture incubator. Two motors are used to rotate the plate, each on a different axis. The 3D clinostat rotates linearly in the clockwise and counter-clockwise directions, and its speed range is from 1 to 10 rpm. Cells in the 3D clinostat trial group were placed on the 3D clinostat and subjected to microgravity conditions for 24, 48, or 72 h. Cells in the static trial group were maintained in a normal incubator at 1 g for the same periods of time. The rotation speed was set to 2 rpm (1 × 10^−3^ g of microgravity), and the entire system was placed in a cell culture incubator.

### 4.3. Cell Viability Assays

Cell viability was measured using Cell Counting Kit-8 (Dojindo Laboratories, Tokyo, Japan). Cells were maintained in 96-well plates (Corning Inc., Corning, NY, USA) with a density of 5 × 10^3^ cells per well in the presence or absence of melatonin (100, 200 nM). After clinostat treatment, cells were added with Cell Counting Kit-8 reagents, incubated in a humidified incubator for 30 min, and absorbance with 450 nm was measured using a plate reader (Molecular Device, Sunnyvale, CA, USA). The % viability was calculated as the absorbance of the melatonin-treated sample/control absorbance ×100.

### 4.4. Western Blot Analysis

MC3T3-E1 cells were collected, washed twice with chilled phosphate buffered saline (PBS), and then resuspended in 20 mM Tris-HCl buffer (pH 7.4) including protease inhibitors (0.1 mM phenylmethylsulfonyl fluoride, 5 µg/mL aprotinin, 5 µg/mL pepstatin A, and 1 µg/mL chymostatin) and phosphatase inhibitors (5 mM Na_3_VO_4_ and 5 mM NaF). Cell lysate was prepared using buffer (150 mM NaCl, 1% NP-40, 50 mM Tris-HCl, pH 7.4, 0.1 mM phenylmethylsulfonyl fluoride, 5 µg/mL aprotinin, 5 µg/mL pepstatin A, 1 µg/mL chymostatin, 5 mM Na3VO4, and 5 mM NaF), and were centrifuged at 13,000× *g* for 10 min at 4 °C to remove cell debris. The protein concentration was determined using the BCA assay (Sigma-Aldrich). Total proteins (40 µg) or media (20 µL) were electrophoresed by Tris-glycine SDS polyacrylamide gels. These proteins were transferred onto a polyvinylidene difluoride (PVDF) membrane (Millipore Corp., Bedford, MA, USA). The membrane was blocked in 5% dry milk in Tris-buffered saline containing Tween-20 (TBS-T; 20 mM Tris, 150 mM NaCl, pH 7.5 containing 0.1% Tween 20) at room temperature for 1 h, washed three times with TBS-T, and then was incubated with antibodies (diluted as indicated in the brackets) directed against the following proteins: LC3 (Cell Signaling Technology, Beverly, MA, USA); IRE1α (Santa Cruz Biotechnology, Santa Cruz, CA, USA); p-PERK (Cell Signaling Technology); GRP78/BiP (Cell Signaling Technology); p-mTOR (Ser2448) and mTOR (Cell Signaling Technology); p-Akt (Ser473) and Akt (Cell Signaling Technology); Cu/Zn-SOD (Santa Cruz Biotechnology); Mn-SOD (Santa Cruz Biotechnology); catalase (Santa Cruz Biotechnology); Bcl-2 (Santa Cruz Biotechnology); Bax (Santa Cruz Biotechnology); Bid (Cell Signaling Technology); and Actin (Assay Designs, Ann Arbor, MI, USA). Immunoreactive proteins were exposed to X-ray film. These films were scanned, and optical densities of protein bands were analyzed using ImageJ analysis software (version 1.37; Wayne Rasband, NIH, Bethesda, MD, USA).

### 4.5. Immunofluorescence Staining

MC3T3-E1 cells cultured on culture slides (BD Falcon Labware, REF 354108) were fixed in methanol at −20 °C for 3 min. Cells were washed with PBS, blocked with 10% bovine serum albumin (Sigma-Aldrich) in PBS for 10 min, and incubated with primary antibody, Lamp2 (Cell Signaling Technology) and LC3 (Cell Signaling Technology), in blocking buffer at 4 °C overnight. Cells were hybridized with secondary antibodies, Alexa 594 (red)-conjugated anti-rabbit IgG (Vector Laboratories Inc., Burlingame, CA, USA) and fluorescein isothiocyanate (green)-labeled anti-mouse IgG (Jackson ImmunoResearch Laboratories, West Grove, PA, USA), for 1 h at room temperature. The coverslips on glass slides were mounted using Vectashield mounting medium (Vector Laboratories Inc.). Cells were observed under a Leica TCS SP5 confocal microscope (Leica, Microsystems CMS GmbH, Wetzlar, Germany). Cells were stained with 4,6-diamidino-2-phenylindole (DAPI, Santa Cruz Biotechnology) for 10 min. Immunofluorescence quantities of both LC3 and Lamp2 indicate the level of autophagosomes or autophagy.

### 4.6. Statistical Analysis

Data were performed statistically to determine significant differences (*p* < 0.05) using one-way analysis of variance (ANOVA), followed by Tukey’s test for multiple comparisons test. Data are expressed as means ± SD of at least three independent experiments. All the data were analyzed using the graphing analysis software (Prism version 4.0; Graph Pad Software Inc., San Diego, CA, USA).

## 5. Conclusions

In conclusion, these results indicate that treatment with melatonin suppresses clinostat-induced autophagy through increases in phosphorylation of ERK/Akt/mTOR. Moreover, melatonin appears to be a potential therapeutic agent for bone loss or osteoporosis associated with microgravity (Figure 7).
